# Effects of dietary energy and lipase levels on nutrient digestibility, digestive physiology and noxious gas emission in weaning pigs

**DOI:** 10.5713/ajas.18.0087

**Published:** 2018-05-31

**Authors:** J. B. Liu, S. C. Cao, J. Liu, J. Pu, L. Chen, H. F. Zhang

**Affiliations:** 1 School of Life Science and Engineering, Southwest University of Science and Technology, Mianyang 621010, China; 2 State Key Laboratory of Animal Nutrition, Institute of Animal Sciences, Chinese Academy of Agricultural Sciences, Beijing 100193, China

**Keywords:** Digestibility, Enzyme Activity, Growth Performance, Lipase, Weaning Pigs

## Abstract

**Objective:**

This study was conducted to evaluate the effect of dietary energy and lipase supplementation on growth performance, nutrient digestibility, serum profiles, intestinal morphology, small intestinal digestive enzyme activities, biochemical index of intestinal development and noxious gas emission in weaning pigs.

**Methods:**

A total of 240 weaning pigs ([Yorkshire×Landrace]×Duroc) with an average body weight (BW) of 7.3±0.12 kg were used in this 28-d experiment. Weaning pigs were randomly allocated to 4 dietary treatments in a 2×2 factorial arrangement with 2 levels of energy (net energy = 2,470 kcal/kg for low energy diet and 2,545 kcal/kg for basal diet) and 2 levels of lipase (0 and 1.5 U/g of lipase) according to BW and sex. There were 6 replications (pens) per treatment and 10 pigs per pen (5 barrows and 5 gilts).

**Results:**

Weaning pigs fed the low energy diet had lower (p<0.05) gain-to-feed ratio (G:F) throughout the experiment, apparent digestibility of dry matter, nitrogen, ether extract, and gross energy during d 0 to 14, average daily gain during d 15 to 28, lipase activity in duodenum and ileum and protein/DNA in jejunum (p<0.05), respectively. Lipase supplementation had no effect on growth performance but affected apparent nutrient digestibility (p<0.05) on d 14 and enhanced lipase activity in the duodenum and ileum and protease activity in duodenum and jejunum of pigs (p<0.05) fed the low energy diet. Lipase reduced serum low-density lipoprotein cholesterol (LDL-C) and triglyceride (TG), NH_3_ production (p<0.05) from the feces.

**Conclusion:**

The low energy diet decreased G:F throughout the experiment and nutrient digestibility during d 0 to 14 as well as lipase activity in duodenum and ileum. Lipase supplementation increased nutrient digestibility during d 0 to 14 and exerted beneficial effects on lipase activity in duodenum and ileum as well as protease activity in duodenum and jejunum, while reduced serum LDL-C, TG and fecal NH_3_.

## INTRODUCTION

Various lipids, including animal fats and plant oils, have been added in the swine diets to improve energy concentrations, which are one of the main energy sources for animals and have higher available energy than other nutrients [1]. The sources, type, supplementation concentration, the composition of the diets and the age may influence the lipid digestion and absorption in weaning pig [2]. In addition, due to the sudden changes of nutrition, environment and physiology, weaning might lead to the weaned stress syndrome. Immature digestive tract and endogenous secretion systems in the early weaning pigs can result in insufficient digestive enzyme activities and gastrointestinal dysbacteriosis [3]. The insufficient secretion of endogenous lipase caused by the aforementioned reasons is likely to restrict the digestion and utilization of lipids in early weaning pigs.

The level of energy in the diet greatly influences the intake of all other nutrients. It is usually accepted that high energy diet is produced for weaning pigs by the addition of lipids. The gain-to-feed ratio (G:F) was increased from a summary which reviewed 92 studies about lipid effects on growth performance in the weaning pigs [4]. Furthermore, the addition of lipid in weaning pigs’ diets can improve the G:F and palatability [5]. One of the approaches to overcome poor lipid digestibility is the supplementation of diets offered to the weaning pigs with appropriate exogenous lipase [6]. Lipase (EC3.1.1.3), triacylglyceryl acylase, is an enzyme involved in catalysis and the hydrolysis of lipids. Compared with the studies about phytase and non-starch polysaccharide enzyme, the application of adding dietary lipase is rare in weaning pigs. Lipase (500 mg/kg) did not affect apparent ileal digestibility (AID) of fat but improved AID of dry matter (DM) and energy as well as the apparent total tract digestibility (ATTD) of DM, organic matter (OM), crude protein (CP), ash and energy in cannula pigs from 20 to 65 kg [7]. Besides, lipase (200 mg/kg) improved G:F but had no effect on average daily gain (ADG) or average daily feed intake (ADFI) in weaning pigs [8].

As the inconsistent results and lack of studies, especially the digestive physiology, this objective of this study was to evaluate the effect of dietary energy and lipase levels on growth performance, nutrient digestibility, serum profiles, intestinal morphology, small intestinal digestive enzyme activities, biochemical index of intestinal development and noxious gas emission in weaning pigs. Furthermore, several studies have identified the positive effects of exogenous enzymes in poultry fed low energy diets [9,10]. However, reports about effect of lipase on weaning pigs are very limited. Thus, we hypothesized that lipase might have a beneficial effect in weaning pigs when they were fed a low energy diet.

## MATERIALS AND METHODS

### Sources of lipase

The acidic heat-resistant lipase from microbial source used in this study was manufactured by Habio (Mianyang, China) and guaranteed to contain 10,000 U/g with its commercial name of Lipozyme. One unit of lipase is the enzyme activity required to release 1 μmol of free fatty acid from tricaprylin substrate per min at 40°C and pH 5.5.

### Animals, housing, and treatments

All animals received humane care as outlined in the Guide for the Care and Use of Experimental Animals (Southwest University of Science and Technology, Animal Care Committee). A total of 240 pigs ([Landrace×Yorkshire]×Duroc), weaned at 21 d of age with an average initial body weight (BW) of 7.3±0.12 kg were assigned to 1 of 4 dietary treatments in a 2×2 factorial arrangement. The treatments consisted of 2 levels of energy (net energy = 2,470 kcal/kg for low energy diet and 2,545 kcal/kg for basal diet) and 2 levels of lipase (0 and 1.5 U/g) according to the BW and sex. There were 6 replications (pens) per treatment and 10 pigs per pen (5 barrows and 5 gilts). The lipase was added at the expense of corn (0.015%). The diets were pellets and formulated to provide all the nutrients to meet or exceed NRC requirements (Table 1) [11]. The experiment lasted for 28 d.

All the pigs were housed in an environmentally controlled nursery facility with slatted plastic flooring and a mechanical ventilation system. The environmental temperature was maintained at 30°C for the first wk of the experiment, and was then reduced by 1°C per week over the next three wks. Each pen (2×2.5 m) was provided with a stainless steel feeder and one nipple waterer, which allowed *ad libitum* access to feed and water throughout the experiment.

### Experimental procedures, sampling, and analysis

The pigs were weighed individually on d 1, d 14, and 28 of the experiment. Feed consumption per pen was also assessed on d 14 and 28 of the experiment. The ADG, ADFI, and G:F were calculated. The pigs were visually observed daily in the morning for 3 to 4 h and incidence of diarrhea was recorded for each piglet based on the method described previously [12,13]. Diarrhea score of each pig was assessed visually each day in the entire experimental period with a score from 1 to 5 (1 = normal feces, 2 = moist feces, 3 = mild diarrhea, 4 = severe diarrhea, and 5 = watery diarrhea). Diarrhea index was then calculated as the percentage of pigs with a diarrhea score of 3 and greater in the total pigs of each group.

During d 8 to 14 and 22 to 28, chromic oxide (0.3%) was added to all the diets as an indigestible index for the determination of apparent nutrient digestibility. On d 13 to 14 and the last 2 d of the experiment, fecal samples (at least 0.25 kg) were collected from at least 2 pigs randomly from each pen via rectal massage, then pooled within the pens. All the feed and fecal samples were stored at −20°C until further analysis. Before chemical analysis, fecal samples were dried at 57°C for 72 h, after which they were ground to pass through a 1-mm screen. Experimental feeds were analyzed for CP, calcium and phosphorus and ether extract (EE) in accordance with AOAC procedures [14]. The amino acid profile of diets was analyzed by HPLC (Hitachi L-8800 Amino Acid Analyzer, Tokyo, Japan) as described [15]. All samples were hydrolyzed at 110°C for 24 h in 6 *N* HCl before analysis. Methionine was analyzed as Met sulfone after cold performic acid oxidation overnight before hydrolysis. The feces were analyzed for DM, N, and EE according to AOAC [14]. Chromium was analyzed via UV absorption spectrophotometry (Shimadzu, UV-1201, Kyoto, Japan) [16]. The gross energy (GE) was determined by measuring the heat of combustion in the samples using a Parr 6100 oxygen bomb calorimeter (Parr instrument Co., Moline, IL, USA). The ATTD of DM and N was calculated using indirect-ratio methods. The gross energy in the feed and feces was determined using a calorimeter (Mode1241, Parr Instrument Co., USA). The nutrient digestibility was calculated using the following formula: digestibility (%) = (1−[{**N_f_**×**C_d_**}/{**N_d_**×**C_f_**}]) ×100 [17], where N_f_ is the nutrient concentration in feces (% DM), N_d_ the nutrient concentration in diet (% DM), C_d_ the chromium concentration in diet (% DM), and C_f_ the chromium concentration in feces (% DM).

On d 28, 4 pigs (2 barrows and 2 gilts) were randomly selected from each pen and blood samples were collected from the jugular vein into a sterile syringe and stored at 4°C. Blood samples were then centrifuged at 3,000×*g* for 15 min at 4°C and serum was separated. The concentrations of glucose (GLU), total protein (TP), albumin (ALB), and activities of alanine aminotransferase (ALT), aspartate aminotransferase (AST), and alkaline phosphatase (ALP) in the serum were measured with an automatic biochemical analyzer (Model 7020; Hitachi, Tokyo, Japan) using the assay kits (Shanghai Shensuo Youfu Medical Diagnostics Co. Ltd., Shanghai, China). The total cholesterol (TC), high-density lipoprotein cholesterol (HDL-C), low-density lipoprotein cholesterol (LDL-C) and triglyceride (TG) in the serum samples were analyzed with an autoanalyzer (Automatic Biochemical Analyzer, RA-1000; Bayer Corp., Tarrytown, NY, USA) using colorimetric methods [1].

The samples of small intestine tissues (approximately 3 cm from duodenum, jejunum and ileum, respectively) were collected on d 28 (2 pigs with average BW per pen) for the determination of small intestinal morphology and digestive enzyme activities. The segment approximately 15 cm away from the pyloric junction was considered as the duodenum, that 55 cm away from the pyloric junction was considered the jejunum, and a distal segment approximately 15 cm proximal to the ileocecal junction was considered the ileum [18]. The tissues from duodenum, jejunum and ileum, respectively, were cleaned with saline and then fixed in 10% neutral formalin. The fixed tissues were trimmed, embedded in paraffin for mucosal morphology and integrity. Intestinal morphological measurements included the following 3 indices: villus height (VH), crypt depth (CD), and VH/CD. These indexes were quantified as previously described [19]. Mean values of VH, CD, and their ratio within each segment were calculated.

The digesta samples from each section were subsequently collected by massaging the tract from proximal and distal ends and stored immediately at −20°C. For the analysis, the digesta samples were thawed at room temperature and homogenized in 4 volumes of ice-cold 0.9% sodium chloride solution. The homogenate was centrifuged at 13,800×*g* for 20 min at 4°C and the supernatant was analyzed for amylase, protease, and lipase activities [20]. The activities of amylase, protease and lipase were determined using the method described [21,22], respectively. All determinations were performed in duplicates.

The remaining duodenum, jejunum, and ileum were thawed on an ice tray and then weighed. After weighing, the samples were washed and cleaned by double steaming water, then dried with filter paper. The intestinal mucosa was collected by cover glass plate with the addition of 2 mmol/L TrisHCl (pH = 7.1) at 4°C, and then treated by the high-speed tissue homogenate slurry machine and centrifuged at 4,000×*g* for 30 min and the supernatant was separated. The DNA and RNA content in the supernatant of the duodenum, jejunum, and ileum was extracted and determined [23]. The protein content, protein/DNA, protein/RNA, and RNA/DNA were analyzed as described [24].

At the end of the experiment, fresh fecal samples (at least 0.25 kg) were collected randomly from at least 2 pigs in each pen every afternoon. Then these samples were stored in 2.6-L sealed plastic boxes in duplicate and fermented for 48 h at 32°C. Fecal NH_3_ and H_2_S concentrations were determined. After the fermentation period, a Gas Detector (GV-100S; Gastec Corp., Kanagawa, Japan) was used for gas detection [25]. In these measurements, the plastic boxes were punctured, and head space air was sampled approximately 2 cm above the samples at a rate of 100 mL/min. Concentrations of NH_3_ and H_2_S were measured within the scope of 5 to 100 ppm (No.3 La, detector tube; Gastec Corp., Japan) and 2 to 20 ppm (No. 4 LL, detector tube; Gastec Corp., Japan), respectively.

### Statistical analysis

The data were analyzed as a 2×2 factorial treatment arrangements using the general linear model procedure of SAS (SAS Inst. Inc., Cary, NC, USA) with the pen being considered as the experimental unit [26]. The model utilized included the main effects of energy and lipase, as well as the interaction between energy and lipase. Variability in the data was expressed as the standard error means and a probability level of p<0.05 was statistically significant. The level of 0.05≤p<0.10 was tendency.

## RESULTS

### Growth performance

The G:F was higher (p<0.05) for pigs offered the basal diet in all periods investigated and unaffected by lipase supplementation (Table 2). The ADG was significantly higher (p<0.05) for pigs fed the basal diet in d 15 to 28 but unaffected in the other periods investigated or by lipase supplementation. The ADFI was not significantly affected by diet or lipase in any period but lipase did tend to reduce (p = 0.05) the diarrhea index.

### Nutrient digestibility

On d 14, the ATTD of DM, N, EE, and GE was increased (p< 0.05) in weaning pigs fed basal diets and lipase supplemented diets (Table 3). However, the ATTD of DM, N, EE, and GE was not affected by energy concentration and lipase on d 28. No interaction between energy concentration and lipase was observed on nutrient digestibility during overall period in this study.

### Serum profiles

The concentrations of LDL-C and TG were decreased (p<0.05) by lipase supplementation (Table 4). However, the ALT, AST, TP, ALB, ALP, GLU, HDL-C, and TC concentrations were not influenced by either energy concentration or lipase. No interaction between energy concentration and lipase was observed on serum profiles.

### Small intestinal digestive enzyme activities

Weaning pigs fed lipase supplemented diets showed higher lipase activity (p<0.05) in duodenum and ileum (Table 5), while lipase supplementation increased protease activity in duodenum and jejunum (p<0.05). No effects were observed in amylase among dietary treatments. There was interaction in both duodenum and ileum lipase activity between energy concentration and lipase (p<0.05).

### Small intestinal morphology and biochemical index of intestinal development

Dietary treatments did not affect small intestinal morphology except VH/CD (Table 6). Weaning pigs fed lipase diets tended to a lower (p = 0.08) level of protein/RNA in duodenum (Table 7). Feeding basal diets increased (p<0.05) the level of protein/DNA in jejunum, while tended to reduce (p = 0.07) protein/DNA in ileum. The supplementation of lipase had the tendency to decrease (p = 0.08) protein/DNA in ileum. No differences were observed in protein, DNA, RNA, or RNA/DNA among dietary treatments. No interaction between energy concentration and lipase was observed on intestinal morphology and biochemical index of intestinal development in this study.

### Noxious gas contents

The fecal NH_3_ was reduced (p<0.05) in weaning pigs fed lipase supplemented diets (Table 8). Fecal H_2_S concentration was not affected by dietary energy concentration and lipase. There was no interaction between energy concentration and lipase on noxious gas contents.

## DISCUSSION

### Effect of energy

Pigs eat to satisfy a demand for energy so that the actual amount of feed consumed depends on the energy density of the diet [4]. Our results showed a positive effect of dietary energy content on feed efficiency. Higher G:F in weaning pigs fed basal diets throughout this study was in agreement with previous study [4], which summarized 92 studies about lipid effects on growth performance in the weaning pigs (5 to 20 kg) and observed feed efficiency was improved. Moreover, it was reported that the predominant effects of lipid at this stage of production increased feed efficiency and palatability [5]. The increased energy concentration was achieved by the greater addition of lard, while the calorie:protein ratio was not maintained in this study. Protein is the nutrient that is most frequently adjusted as energy density is changed, and the same logic can be applied to all nutrients. A recent study found a consistent decrease in ADFI (average −50 g/d) with the addition of lipid in 59 of 92 studies and even more reduction (−70 g/d) in response to constant calorie:protein ratio [4]. Although ADG and ADFI during d 0 to 14 and the overall period were not affected significantly by dietary energy concentrations in this study, there was similar numerical decrease (approximately 9 g/d and 34.5 g/d, respectively), indicating that the reduction in ADFI may be greater than that in ADG. Furthermore, increased ADG of weaning pigs fed basal diets during d 15 to 28 observed in the current study agreed with previous study [5], which reported that lipid supplementation did not increase growth performance in the first 2 wks after weaning compared with the next 2 wks. This may be due to the decrease of lipase in pancreatic section from d 3 to 7 and the increase from d 7 to 28 after weaning [27]. A recent study also found that growth performance was not affected by dietary energy in weaning pigs during d 28 to 42, whereas weaning pigs fed the basal energy diet exhibited better G:F than those fed the low energy diet during d 43 to 70 [28]. They explained that young pigs may be less sensitive to dietary energy concentration than older pigs [28].

The ATTD of DM, N, EE, and GE on d 14 was increased by the basal diets, which was consistent with previous study [6]. The ATTD of DM, EE, N, and GE was improved with the addition of lard in weaning pigs [29]. Similarly, several studies also identified the negative effects of low energy diets on nutrient digestibility in weaning pigs and growing-finishing pigs [30,31]. Increased ATTD of DM, N, and GE may be attributed to the increased EE digestibility in our study. Besides, the beneficial effects of dietary energy treatments on nutrient digestibility may partially mirror the increased G:F in this study. It is evident that the energy concentration is the main determinant of voluntary feed intake in pigs among nutrients [4]. In our study, there was no difference in ADFI and we supposed that the increased G:F may be due to the increased digestibility.

In our study, we failed to observe the effects of energy concentration on blood profiles, which were partially consistent with the results of previous study, which indicated that energy density did not influence TC, TG, LDL-C, or HDL-C in weaning pigs [6]. Weaning pigs fed basal diets had higher lipase activity in duodenum and ileum in the current study. To our knowledge, there are no reports about the effects of energy on small digestive enzyme activity in pigs, can we can only compare this aspect with one study in broilers. In agreement with our results, broilers fed high energy diet had higher lipase activity in duodenum and ileum than those fed the low energy diet [32]. Dietary treatments had no effect on intestinal morphology in this study. However, several studies observed increased VH and decreased CD in jejunum in weaning pigs fed high energy diets [33,34].

After the formation of the double cells, DNA content was relatively constant, whose level may reflect the change in the cell population. Protein/DNA ratio was typical index of cell size. Protein/RNA ratio may reflect the potential for protein synthesis and actual protein synthesis rate, namely the ability of cells ribosome’s translation into protein. RNA/DNA ratio reflected the organization’s vitality and the efficiency of individual cells [35]. In this study, protein/DNA ratio in ileum tended to be higher in response to the low energy diets, indicating that the intestinal mucosal cells may be enlarged which may increase the absorption area. Notwithstanding, the exact reason was not clear in the current study. The lack of energy effect on biochemical index of intestinal development may partially mirror its influence on intestinal morphology.

Environmental concerns about noxious gas excreted with the feces have been increased in China. Fecal noxious gas was closely related to the feed efficiency, nutrient utilization and intestinal microbiota [36]. Fecal H_2_S did not differ between reduced energy diets and basal diets in the current study, which may be due to the lack of significant differences in both the sulfur composition and sulfur-containing amino acids of diets among the treatments [37]. Furthermore, the lack of energy effect on N digestibility on d 28 in this study may mirror the fecal NH_3_. Similarly, fecal NH_3_ was not affected by energy concentration in growing pigs [38].

### Effect of lipase

Our results showed that lipase supplementation had no effect on growth performance but affected apparent nutrient digestibility on d 14 and markedly enhanced lipase activity in the duodenum and ileum of pigs offered the low energy diet but not the basal diet. Feed intake and subsequent growth rate may be restricted by the intestinal capacity of the newly weaning pigs, therefore high energy diets might be used in weaning pigs to express their genetic potential. To achieve these requirements, great amount of animal fats and vegetable oils are usually added to weaning pig diets to increase their energy concentration. It was well known that there might be a transition phase in lipid digestibility during the initial 3-wk postweaning period for pigs weaned at 21 d of age. The addition of lipase (2 U/g) did not affect ADG or ADFI, but improved G:F in weaning pigs fed diets containing 1% to 2% soybean oil [6]. Supplemental lipase (1 U/g) increased the ADG and the G:F in weaning pigs [39]. Unfortunately, we failed to observe positive effects of lipase supplementation on growth performance in this study, which was consistent with previous study which reported 6.5 U/g lipase had no effect on growth performance in weaning pigs fed diets containing 2.5% soybean oil [6]. In agreement with previous study, lipase supplementation tended to reduce diarrhea index in this study, which may be due to the released medium-chain fatty acids [39]. The addition of lipase may release the fatty acids from fat, including some medium-chain fatty acids, which can inhibit the growth of harmful microorganisms [5]. The potential reasons for the differences between current study and previous work may be due to the different supplemental concentration, lipase source and lipid type.

Supplemental lipase (2.5 U/g) increased AID of DM and GE as well as ATTD of DM, OM, CP, ash and GE in cannula pigs fed 4% animal fat from 20 to 65 kg, whereas did not affect AID of EE [40]. Similarly, our results found that lipase improved ATTD of DM, N, EE, and GE on d 14 in the present study. A study failed to observe the increase in ATTD of EE in weaning pigs fed lipase-supplemented diets (2 U/g), but ATTD of DM, N, and GE tended to be improved [6]. From researches mentioned above, we hypothesize that the inconsistent results of growth performance and nutrient digestibility were due to diet fat source and inclusion rate and specific lipase used and the amount added. Little is known about the effects of lipase on growth performance and nutrient utilization so that further study is needed.

Lipase supplementation (2 U/g) decreased serum TG, TC, LDL-C, and HDL-C in weaning pigs [6]. In our study, lipase also reduced serum LDL-C and TG in pigs offered both diets, which was consistent with previous study, which indicated lipase addition (200 mg/kg) reduced TG and LDL-C in weaning pigs [39]. Serum TP was not affected in this study, while a study found that serum TP was increased by lipase supplementation [40]. Due to there being few reports about the effects of lipase on blood profiles in pigs, we can compare this aspect only with emulsifier in weaning pigs. Several studies indicated that TG was reduced by lipid plus emulsifier in weaning pigs [1,41]. Lipase supplementation was conducive to the body’s fat metabolism, played a role in strengthening the fat mobilization and inhibition of fat synthesis, and promoted the body’s use of fat, thereby reducing the serum TC and TG content [6]. The mechanism of how lipase affects TG or TC is still unclear. We hypothesized that TG and LDL-C can be reduced at a later period after weaning by lipase addition.

Lipase supplementation (200 mg/kg) to weaning pigs’ diets improved the activities of lipase and amylase in duodenum [39]. Our results showed that lipase addition increased the activities of lipase and protease in duodenum. Furthermore, protease activity in jejunum and lipase activity in ileum was increased by lipase addition in the current study. However, the improvement in small intestinal digestive enzymes did not exert beneficial effects on nutrient digestibility in the later period. Besides, enzyme complex containing amylase, protease and xylanase (100 to 300 mg/kg) enhanced the activities of amylase, lipase and protease in the small intestine in weaning pigs [42].

The supplemental lipase (200 mg/kg) increased VH, VH/CD, and decreased CD in duodenum and jejunum in weaning pigs [39]. Notwithstanding, we only observed increased VH/CD in jejunum. In this study, protein/RNA ratio in duodenum and protein/DNA tended to be decreased. However, the exact reason was not clear in the current study and conclusions cannot be drawn just from this study.

The NH_3_ in manure is significant source of pollution in pig production that must be reduced. Previous studies have reported that inclusion of appropriate exogenous enzymes to pig diets may alter the manure emission of NH_3_ [43,44]. In the present experiment, lipase supplementation reduced NH_3_ production from the feces of pigs offered both diets, indicating that N utilization was improved by lipase. Contrary to the present results, a study reported an increased proportion of NH_3_ in finisher pigs fed barley-based diets supplemented with exogenous enzymes (glucanase and xylanase) compared with pigs fed diets that were not supplemented with exogenous enzymes [44]. Dietary supplementation of 500 mg/kg of enzyme blend (mannanase, amylase, and protease) to corn-soybean meal-based or complex diets did not affect fecal emission of NH_3_ in growing pigs [38]. The discrepancy may be due to the different types of cereals and sources of exogenous enzyme. It has been reported that NH_3_ emission from pigs’ manure was influenced by cereal type in diets and source of exogenous enzymes [43]. To clarify these differences in the results, more research is needed to further explore the influence of lipase on noxious gas emission in weaning pigs.

### Interactive effect of energy and lipase

Unexpectedly, in our results lipase did not exert a positive effect on any criteria measure in weaning pigs when they were fed low energy diet. We only observed the interactive effect of energy concentration and lipase on lipase activity in duodenum and ileum, although there may be tendency in G:F. Further research is required to elucidate the key factors involved.

## CONCLUSION

Considering the data obtained herein and the above discussion, it can be concluded that weaning pigs fed basal diets had higher G:F throughout the experiment, ATTD of DM, N, EE, and GE during d 0 to 14, ADG during d 15 to 28, lipase activity in duodenum and ileum and protein/DNA in jejunum. Furthermore, lipase supplementation had no effect on growth performance but affected apparent nutrient digestibility at d 14 and markedly enhanced lipase activity in the duodenum and ileum of pigs fed the low energy diet but not the standard diet and reduced NH_3_ production from the feces of pigs offered both diets. Lipase also reduced serum LDL-C and TG in pigs offered both diets. Interaction was observed in both duodenum and ileum lipase activity between energy concentration and lipase.

## Figures and Tables

**Table 1:** Diet composition (as-fed basis)

Items	Low energy diet	Basal diet
Ingredients (%)		
Corn	51.85	49.05
Extruded corn	10.00	10.00
Soybean meal (CP, 48%)	20.35	21.55
Fish meal (CP, 66%)	4.00	4.00
Whey	4.40	4.40
Lard	3.00	5.00
Plasma powder	1.93	1.53
Limestone	0.55	0.55
Dicalcium phosphate	1.52	1.52
NaCl	0.20	0.20
L-Lys·HCl (78.8%)	0.30	0.30
DL-methionine (99%)	0.60	0.60
L-threonine (98.5%)	0.15	0.15
Choline chloride (60%)	0.10	0.10
Zinc oxide (80%)	0.25	0.25
Acidifier	0.50	0.50
Vitamin premix1)	0.10	0.10
Trace mineral premix2)	0.20	0.20
Analyzed composition		
NE (kcal/kg)3)	2,470	2,545
CP (%)	20.95	20.92
Ether extract (%)	3.36	5.79
Lys (%)	1.40	1.42
Met (%)	0.64	0.63
Ca (%)	0.86	0.84
Total P (%)	0.76	0.77

CP, crude protein; NE, net energy.

1) Provided per kilogram of complete diet: vitamin A, 11,025 IU; vitamin D_3_, 1,103 IU; vitamin E, 44 IU; vitamin K, 4.4 mg; riboflavin, 8.3 mg; niacin, 50 mg; thiamine, 4 mg; d-pantothenic, 29 mg; choline, 166 mg; biotin 0.09 mg; vitamin B_12_, 33 μg.

2) Provided per kilogram of complete diet: Cu (as CuSO_4_·5H_2_O), 12 mg; Zn (as ZnSO_4_), 85 mg; Mn (as MnO_2_), 8 mg; I (as KI), 0.28 mg; Se (as Na_2_SeO_3_·5H_2_O), 0.15 mg.

3) Calculated according to NRC [11].

**Table 2:** Effects of dietary energy and lipase levels on growth performance in weaning pigs1)

Items	LED2)		BD		SEM	p-value3)		
		
	−Lipase	+ Lipase	−Lipase	+ Lipase		Energy	Lipase	Energy×lipase
d 0 to 14								
ADG (g)	355	373	371	379	12	0.25	0.14	0.42
ADFI (g)	488	483	446	456	16	0.17	0.22	0.66
G:F	0.73	0.77	0.83	0.83	0.01	0.03	0.40	0.07
d 15 to 28								
ADG (g)	453	480	511	517	13	0.04	0.38	0.52
ADFI (g)	724	713	705	710	19	0.24	0.37	0.50
G:F	0.63	0.67	0.73	0.73	0.01	0.02	0.12	0.15
d 0 to 28								
ADG (g)	404	426	441	448	11	0.19	0.12	0.62
ADFI (g)	606	598	575	583	16	0.73	0.30	0.69
G:F	0.67	0.71	0.77	0.77	0.01	0.03	0.67	0.06
Diarrhea index (%)	6.90	4.59	6.46	4.15	0.52	0.30	0.05	0.51

SEM, pooled standard error of the means; ADG, average daily gain; ADFI, average daily feed intake; G:F, gain-to-feed ratio; NE, net energy.

1) ADG mean represents 6 pens (n = 6/group) and feed consumption mean represents 6 pens (n = 6/group).

2) LED, low energy diet (NE = 2,470 kcal/kg) with or without 1.5 U/g of lipase; BD, basal diet (NE = 2,545 kcal/kg) with or without 1.5 U/g of lipase.

3) Energy, energy effect; Lipase, lipase effect; Energy×lipase, interaction between energy and lipase.

**Table 3:** Effects of dietary energy and lipase levels on apparent fecal digestibility of nutrients in weaning pigs1)

Items	LED2)		BD		SEM	p-value3)		
		
	−Lipase	+Lipase	−Lipase	+Lipase		Energy	Lipase	Energy×lipase
d 14								
DM	78.8	79.9	80.1	81.2	0.2	0.03	0.04	0.78
N	78.9	80.6	80.8	82.7	0.3	0.04	0.03	0.56
EE	64.5	65.7	67.4	66.1	0.5	0.02	0.04	0.08
GE	78.3	80.0	80.1	81.6	0.2	0.03	0.02	0.56
d 28								
DM	78.7	80.2	79.6	80.0	0.3	0.34	0.08	0.43
N	79.8	82.1	79.7	80.4	0.5	0.27	0.19	0.57
EE	66.6	67.4	66.8	67.9	0.6	0.17	0.23	0.63
GE	79.9	81.3	80.1	80.6	0.3	0.72	0.31	0.69

SEM, pooled standard error of the means; DM, dry matter; N, nitrogen; EE, ether extract; GE, gross energy; NE, net energy.

1) Each mean represents 6 pens (n = 6/group).

2) LED, low energy diet (NE = 2,470 kcal/kg) with or without 1.5 U/g of lipase; BD, basal diet (NE = 2,545 kcal/kg) with or without 1.5 U/g of lipase.

3) Energy, energy effect; Lipase, lipase effect; Energy×lipase, interaction between energy and lipase.

**Table 4:** Effects of dietary energy and lipase levels on serum profiles in weaning pigs1)

Items	LED2)		BD		SEM	p-value3)		
		
	−Lipase	+Lipase	−Lipase	+Lipase		Energy	Lipase	Energy×lipase
ALT (U/L)	86.4	78.5	85.4	83.1	4.2	0.21	0.11	0.14
AST (U/L)	74.9	76.0	77.8	82.1	3.8	0.13	0.34	0.25
TP (g/L)	59.2	59.3	61.7	59.5	3.1	0.36	0.23	0.59
ALB (g/L)	34.2	33.3	35.5	35.3	2.0	0.43	0.28	0.67
ALP (U/L)	355	384	381	349	19	0.22	0.14	0.11
GLU (mmol/L)	5.7	5.7	5.5	5.4	1.0	0.34	0.61	0.30
HDL-C (mg/dL)	31.1	30.4	31.0	29.5	3.1	0.66	0.53	0.76
LDL-C (mg/dL)	59.0	46.8	64.2	45.3	3.9	0.19	0.03	0.58
TG (mg/dL)	59.7	44.3	61.0	44.2	4.0	0.43	0.02	0.53
TC (mg/dL)	89.5	95.6	102.2	100.3	5.2	0.83	0.33	0.80

SEM, pooled standard error of the means; ALT, alanine aminotransferase; AST, aspartate aminotransferase; TP, total protein; ALB, albumin; ALP, alkaline phosphatase; GLU, glucose; HDL-C, high-density lipoprotein cholesterol; LDL-C, low-density lipoprotein cholesterol; TC, total cholesterol; TG, triglyceride; NE, net energy.

1) Each mean represents 6 pens (n = 6/group).

2) LED, low energy diet (NE = 2,470 kcal/kg) with or without 1.5 U/g of lipase; BD, basal diet (NE = 2,545 kcal/kg) with or without 1.5 U/g of lipase.

3) Energy, energy effect; Lipase, lipase effect; Energy×lipase, interaction between energy and lipase.

**Table 5:** Effects of dietary energy and lipase levels on small intestinal digestive enzyme activities in weaning pigs1)

Items	LED2)		BD		SEM	p-value3)		
		
	−Lipase	+Lipase	−Lipase	+Lipase		Energy	Lipase	Energy×lipase
Duodenum								
Amylase (U/g)	300	348	341	352	14	0.24	0.15	0.43
Lipase (U/g)	506	638	628	630	15	0.01	0.02	0.04
Protease (U/g)	5,943	6,607	6,477	6,656	128	0.13	0.04	0.55
Jejunum								
Amylase (U/g)	320	375	370	377	12	0.31	0.15	0.36
Lipase (U/g)	308	327	319	320	12	0.83	0.34	0.80
Protease (U/g)	6,646	7,286	7,172	7,269	109	0.27	0.04	0.19
Ileum								
Amylase (U/g)	294	339	336	332	13	0.18	0.97	0.42
Lipase (U/g)	224	316	323	327	14	0.04	0.03	0.04
Protease (U/g)	5,015	5,507	5,471	5,515	111	0.42	0.25	0.33

SEM, pooled standard error of the means; NE, net energy.

1) Each mean represents 6 pens (n = 6/group).

2) LED, low energy diet (NE = 2,470 kcal/kg) with or without 1.5 U/g of lipase; BD, basal diet (NE = 2,545 kcal/kg) with or without 1.5 U/g of lipase.

3) Energy, energy effect; Lipase, lipase effect; Energy×lipase, interaction between energy and lipase.

**Table 6:** Effects of dietary energy and lipase levels on intestinal morphology in weaning pigs1)

Items	LED2)		BD		SEM	p-value3)		
		
	−Lipase	+Lipase	−Lipase	+Lipase		Energy	Lipase	Energy×lipase
Duodenum								
Villus height (μm)	326	352	337	362	23	0.11	0.15	0.67
Crypt depth (μm)	310	300	326	297	32	0.74	0.17	0.25
VH/CD	1.05	1.17	1.03	1.22	0.05	0.31	0.64	0.87
Jejunum								
Villus height (μm)	328	343	319	367	33	0.72	0.11	0.71
Crypt depth (μm)	286	267	269	280	16	0.12	0.29	0.52
VH/CD	1.15	1.28	1.18	1.31	0.04	0.52	0.04	0.12
Ileum								
Villus height (μm)	300	302	282	315	30	0.39	0.20	0.76
Crypt depth (μm)	254	241	232	231	21	0.83	0.31	0.65
VH/CD	1.18	1.25	1.22	1.36	0.07	0.33	0.28	0.19

SEM, pooled standard error of the means; VH, villus height; CD, crypt depth; NE, net energy.

1) Each mean represents 6 pens (n = 6/group).

2) LED, low energy diet (NE = 2,470 kcal/kg) with or without 1.5 U/g of lipase; BD, basal diet (NE = 2,545 kcal/kg) with or without 1.5 U/g of lipase.

3) Energy, energy effect; Lipase, lipase effect; Energy×lipase, interaction between energy and lipase.

**Table 7:** Effects of dietary energy and lipase levels on the biochemical index of intestinal development in weaning pigs1)

Items	LED2)		BD		SEM	p-value3)		
		
	−Lipase	+Lipase	−Lipase	+Lipase		Energy	Lipase	Energy×lipase
Duodenum								
Protein (mg/g)	54.5	53.3	52.7	51.5	1.9	0.17	0.20	0.64
DNA (mg/g)	5.38	4.36	4.87	4.29	0.45	0.29	0.31	0.60
RNA (mg/g)	0.24	0.26	0.25	0.26	0.01	0.30	0.40	0.68
Protein/DNA	10.13	12.22	10.82	12.00	1.23	0.68	0.17	0.94
Protein/RNA	227	205	211	198	10	0.13	0.08	0.25
RNA/DNA	0.04	0.06	0.05	0.06	0.01	0.48	0.11	0.97
Jejunum								
Protein (mg/g)	49.0	52.0	52.3	53.5	3.9	0.45	0.54	0.65
DNA (mg/g)	6.49	5.92	5.50	5.36	0.38	0.23	0.86	0.99
RNA ((mg/g)	0.40	0.43	0.44	0.44	0.02	0.19	0.86	0.99
Protein/DNA	7.55	8.78	9.51	9.97	0.51	0.04	0.16	0.40
Protein/RNA	123	121	119	122	16	0.23	0.57	0.85
RNA/DNA	0.06	0.07	0.08	0.08	0.01	0.32	0.44	0.68
Ileum								
Protein (mg/g)	72.1	64.2	62.3	60.7	4.2	0.32	0.44	0.72
DNA (mg/g)	8.05	9.16	9.89	10.05	0.48	0.13	0.34	0.48
RNA (mg/g)	0.51	0.50	0.49	0.50	0.01	0.27	0.29	0.87
Protein/DNA	8.96	7.00	6.30	6.03	0.54	0.07	0.08	0.17
Protein/RNA	142	128	127	121	8	0.40	0.52	0.74
RNA/DNA	0.06	0.05	0.05	0.05	0.01	0.18	0.47	0.96

SEM, pooled standard error of the means; NE, net energy.

1) Each mean represents 6 pens (n = 6/group).

2) LED, low energy diet (NE = 2,470 kcal/kg) with or without 1.5 U/g of lipase; BD, basal diet (NE = 2,545 kcal/kg) with or without 1.5 U/g of lipase.

3) Energy, energy effect; Lipase, lipase effect; Energy×lipase, interaction between energy and lipase.

**Table 8:** Effects of dietary energy and lipase levels on noxious gas contents in weaning pigs1)

Items	LED2)		BD		SEM	p-value3)		
		
	−Lipase	+Lipase	−Lipase	+Lipase		Energy	Lipase	Energy×lipase
NH_3_ (mg/kg)	28.4	21.7	27.3	19.5	2.3	0.19	0.04	0.51
H_2_S (mg/kg)	4.1	3.6	4.5	5.3	1.1	0.18	0.96	0.42

SEM, pooled standard error of the means; NE, net energy.

1) Each mean represents 6 pens (n = 6/group).

2) LED, low energy diet (NE = 2,470 kcal/kg) with or without 1.5 U/g of lipase; BD, basal diet (NE = 2,545 kcal/kg) with or without 1.5 U/g of lipase.

3) Energy, energy effect; Lipase, lipase effect; Energy×lipase, interaction between energy and lipase.
